# 802 Increase in Surgical Burn Management During the COVID-19 Pandemic

**DOI:** 10.1093/jbcr/irae036.342

**Published:** 2024-04-17

**Authors:** Sara C Chaker, Mariam Saad, Andrew James, Ricardo Torres-Guzman, Elizabeth L Slater, William Lineaweaver

**Affiliations:** Vanderbilt University Medical Center, Nashville, TN; Vanderbilt University, Nashvile, TN; Vanderbilt University Medical Center, Nashville, TN; Vanderbilt University, Nashvile, TN; Vanderbilt University Medical Center, Nashville, TN; Vanderbilt University, Nashvile, TN; Vanderbilt University Medical Center, Nashville, TN; Vanderbilt University, Nashvile, TN; Vanderbilt University Medical Center, Nashville, TN; Vanderbilt University, Nashvile, TN; Vanderbilt University Medical Center, Nashville, TN; Vanderbilt University, Nashvile, TN

## Abstract

**Introduction:**

The COVID-19 pandemic has had a profound impact on healthcare systems worldwide. This study explores the consequences of the pandemic on pediatric burn management, specifically focusing on whether the pandemic contributed to a surge in the volume of pediatric burns requiring surgical interventions.

**Methods:**

All surgical cases that had a post-operative ICD-10 diagnosis of a burn was collected from the National Surgical Quality Improvement Program Pediatric database. Patient demographics, procedure codes, and postoperative complications was collected.

**Results:**

From 2018 to 2021, there was a total of 872 pediatric burns that required surgical intervention. The median age of the patients was 5.4 years. There was an 8.7% increase in volume of these cases from 2019 to 2020. The overall rate of complications was 8.4%. The predominant surgical approach employed in the majority of cases was the utilization of split-thickness autograft.

**Conclusions:**

There was an increase in pediatric surgical burn treatment volume during the COVID-19 pandemic. Further investigation into the underlying causes and implications of this surge is warranted.

**Applicability of Research to Practice:**

This analysis provides more information on the incidence of pediatric burns managed by surgical treatment and how it was affected by the COVID-19 pandemic.

